# Supplementary material for: The Combination of Oncolytic Virus and Antibody Blockade of TGF-β Enhances the Efficacy of αvβ6-Targeting CAR T Cells Against Pancreatic Cancer in an Immunocompetent Model
Source: Cancers (Basel). 2025 Apr 30;17(9):1534. doi: 10.3390/cancers17091534 (PMC12070938; doi:10.3390/cancers17091534)
Supplement: Supplementary file 1 [file cancers-17-01534-s001.zip › cancers-3512438-supplementary.pdf]

Supplementary Figures

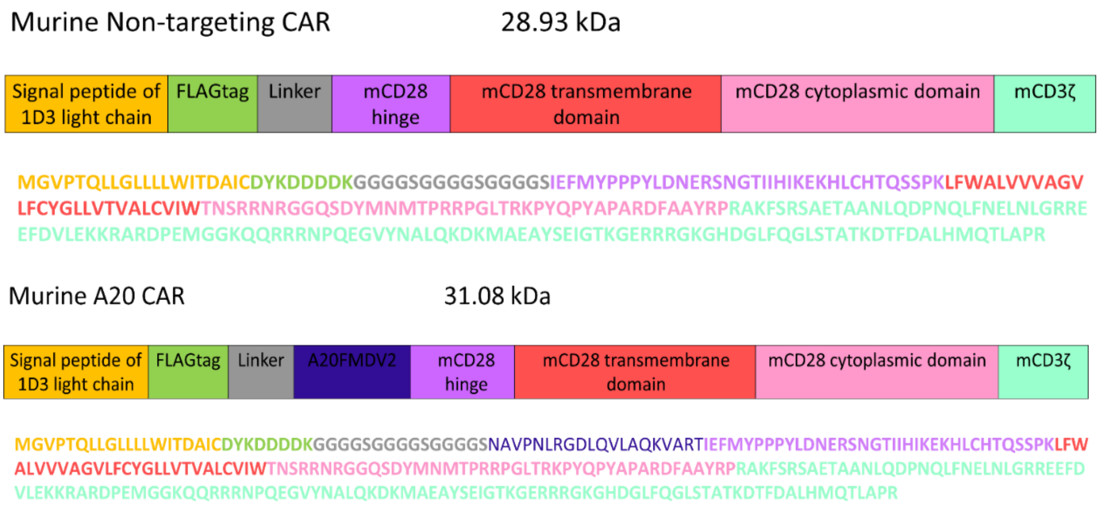

Figure S1. Structure of murine non-targeting CAR and mA20CAR.

Antigen Target	Fluorochrome	Manufacturer	Catalog Number
Anti-Mouse CD8a	PE	BD bioscience	553032
Anti-Mouse CD8a	Brilliant Violet 570	Biolegend	100740
Anti-mouse CD4	Pacific Blue	Biolegend	100428
Anti-mouse CD62L	Brilliant Violet 570	Biolegend	104433
Anti-mouse CD62L	Pacific Blue	BioLegend	104424
Anti-mouse/human CD44	Alexa Fluor 488	Biolegend	103016
Anti-mouse CD45	Brilliant Violet 421	BioLegend	103134
Anti-mouse CD279 (PD-1)	FITC	Biolegend	135214
Anti-mouse/human CD44	Alexa Fluor 488	Biolegend	103016
APC anti-DYKDDDDK Tag	Red Laser (633 nm)	Biolegend	637308
Anti-DYKDDDDK Tag	Alexa Fluor 647	Cell Signaling	15009
His Tag Monoclonal	Alexa Fluor 488	ThermoFisher	MA1-21315-A488

Figure S2. Antibody Panel Used in the Flow Cytometry Assays.

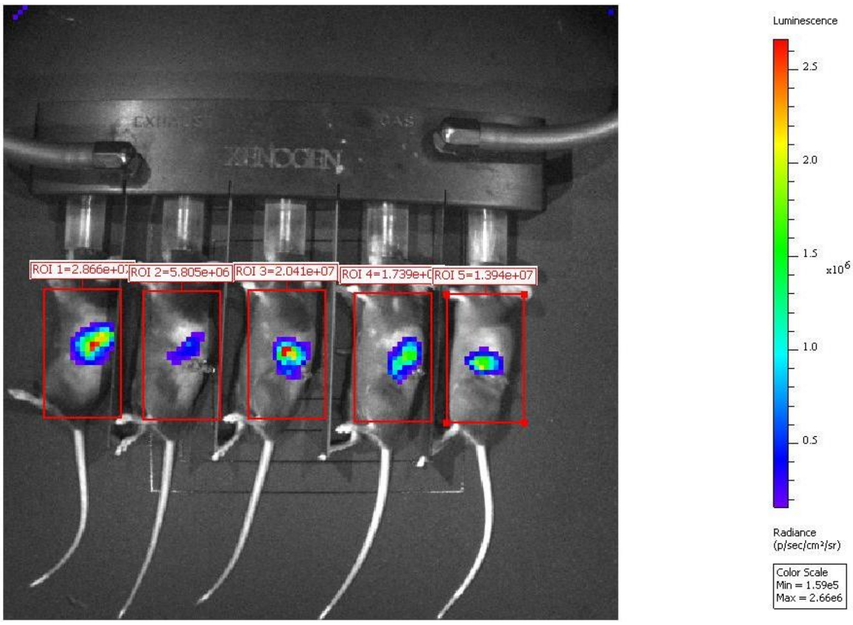

Figure S3. In Vivo Bioluminescence Imaging Using the IVIS Spectrum System. Bioluminescence detected by IVIS imaging. An example of quantifying bioluminescence and calculating ROI values reflecting tumour sizes.
